# Tomatidine relieves neuronal damage in spinal cord injury by inhibiting the inflammatory responses and apoptosis through blocking the NF-κB/CXCL10 pathway activation

**DOI:** 10.3389/fphar.2024.1503925

**Published:** 2024-12-12

**Authors:** Xu Wang, Wei Huang, Hao Sun, Hua Wang, Dongxu Wang, Yongxiang Wang

**Affiliations:** ^1^ The Yangzhou School of Clinical Medicine of Nanjing Medical University, Yangzhou, China; ^2^ Department of Trauma Surgery, Northern Jiangsu People’s Hospital, Yangzhou, China; ^3^ Health Management Center, Northern Jiangsu People’s Hospital, Yangzhou, China; ^4^ School of Grain Science and Technology, Jiangsu University of Science and Technology, Zhenjiang, China

**Keywords:** tomatidine, spinal cord injury, neuronal damage, nuclear factor-κB/C-X-C motif chemokine ligand 10 pathway, apoptosis

## Abstract

**Background:**

Spinal cord injury (SCI) is a neurological disease characterized by high disability and mortality rates. Tomatidine, a natural steroid alkaloid, has been evidenced to have neuroprotective properties. However, the underlying mechanisms of tomatidine in treating SCI remain ambiguous. This study aimed to illustrate the molecular mechanisms of tomatidine in modulating the inflammatory response and promoting functional rehabilitation after SCI.

**Methods:**

Sprague–Dawley (SD) rats were used to construct an *in vivo* SCI model and were intraperitoneally injected with tomatidine (5, 10, or 20 mg/kg) for 7 days, followed by treatment with the nuclear factor-κB (NF-κB) pathway agonist (PMA). In addition, lipopolysaccharide (LPS)-induced PC-12 cells were used to establish an SCI cell model and were stimulated with tomatidine, PMA, or a CXCL10 inhibitor. The pathophysiological changes and neurological function were evaluated using blood-brain barrier (BBB) scoring, water content determination, hematoxylin and eosin (H&E) staining, and TUNEL assay. Levels of inflammatory cytokines, including tumor necrosis factor (TNF)-α, interleukin (IL)-1β, and IL-6, were measured. Cell proliferation, apoptosis, and the expression of C-X-C motif chemokine ligand 10 (CXCL10) were determined. Moreover, the expression of cleaved-caspase 3, caspase 3, CXCL10, p-p65, and p65 were analyzed.

**Results:**

Our data revealed that tomatidine promoted neuronal damage recovery, reduced histopathological changes, elevated cell proliferation, and inhibited the apoptosis and inflammatory factor levels in spinal cord tissues and LPS-induced PC-12 cells. Moreover, tomatidine decreased the expression of CXCL10 *in vitro* and *in vivo*, which was accompanied by the regulation of the NF-κB pathway. However, the NF-κB pathway agonist PMA reversed the protective effect of tomatidine *in vitro*. PMA also enhanced the CXCL10 expression and stimulated the activation of the NF-κB pathway, as demonstrated by the upregulation of phosphorylated p65. The CXCL10 inhibitor had effects similar to tomatidine on cleaved-caspase 3 expression, CXCL10 expression, and the NF-κB pathway.

**Conclusion:**

Tomatidine can alleviate neuronal damage in SCI by inhibiting apoptosis and inflammation through the NF-κB/CXCL10 pathway. Our findings provide a novel therapeutic target and candidate for the treatment of SCI.

## 1 Introduction

Spinal cord injury (SCI) is a central nervous system disease characterized by high morbidity and mortality worldwide (Anjum et al., 2020). More than 17,000 new cases of spinal cord injury occur each year, resulting in motor and neurological deficits for 282,000 people (Hu et al., 2023). Currently, the clinical treatment of spinal cord injury focuses on surgical treatment, pharmacological treatment, complication prevention, and functional rehabilitation (Kjell and Olson, 2016; Sterner and Sterner, 2022). However, all these clinical treatments have shown limited efficacy. Previous research studies revealed that modulating the inflammation and microenvironment after SCI is advantageous for the recovery of neural tissues (Freyermuth-Trujillo et al., 2022). The inflammatory response after SCI is mainly regulated by cytokines, chemokines, and reactive oxygen species secreted by the central nervous system-activated neuroglia and peripheral immune cells (Lin et al., 2021). Therefore, understanding the molecular mechanisms underlying the pathogenesis of SCI is crucial for the discovery of new therapeutic approaches to treat SCI.

Tomatidine, a steroidal alkaloid from the *Solanaceae* family, has been evidenced to have anti-inflammatory, anti-tumorigenic, and anti-osteoporosis effects. A previous study revealed that tomatidine suppressed cell invasion through the negative modulation of gelatinase and inactivation of p38 and extracellular signal-regulated kinase (ERK) (Jeon and Kim, 2019). Yu T. et al. (2020) revealed that tomatidine improved osteoporosis, and one of the mechanisms of its action is achieved by regulating p53. Moreover, tomatidine inhibited oxidative stress and inflammation to improve acute lung injury in mice (Huang et al., 2021). However, the roles and underlying mechanisms of tomatidine in treating SCI have not been fully reported.

Previous investigations have suggested that excessive inflammatory response releases large amounts of pro-inflammatory cytokines that promote neuronal cell apoptosis and further aggravate secondary injury after SCI (Yin et al., 2022). Nuclear factor-κB (NF-κB) is an important inflammatory mediator transcription factor involved in the pathophysiological process of SCI. For instance, Liu et al. (2021) revealed that SARM1 promoted neuroinflammation and suppressed neural regeneration after SCI through the NF-κB signaling pathway. Inflammatory factors include a variety of cytokines and chemokines, such as interleukin (IL)-6, chemokine ligand (CXCL)10, and CXCL13 (Soltani Khaboushan et al., 2022). CXCL10, also known as interferon (IFN)-γ-inducible protein-10, is a member of the CXC chemokine family with potent chemotactic effects on activated T-cells, natural killer cells, and monocytes (Mao et al., 2023; Ozga et al., 2022). CXCL10 was evidenced to promote tumor progression via CXC chemokine receptor 3 (CXCR3) in melanoma (Doron et al., 2019), lung cancer (Hong et al., 2022), and bladder cancer (Nazari et al., 2020). Moreover, a growing number of research studies have demonstrated that CXCL10 is involved in the regulation of neuronal injury after SCI. Qiao et al. (2022) reported that CXCL10 was upregulated in both young and old mice following SCI. However, it has not been reported whether tomatidine can alleviate neuronal damage in spinal cord injury by regulating CXCL10 expression through the NF-κB signaling pathway.

Thus, we hypothesized that tomatidine may improve neuronal damage in spinal cord injury by inhibiting inflammatory responses and apoptosis through the NF-κB/CXCL10 pathway. In our research, *in vitro* and *in vivo* SCI models were used to illustrate the regulatory function of tomatidine in spinal cord tissues of SCI rats and lipopolysaccharide (LPS)-induced PC-12 cells, exploring its underlying mechanisms through the NF-κB/CXCL10 pathway. Our findings may provide new therapeutic targets and candidate for SCI treatment.

## 2 Materials and methods

### 2.1 Animals

Sprague–Dawley (SD, 6-week-old, weight 200–220 g) rats were used for this research. The rats were purchased from Hubei Beiente Biotechnology Co., Ltd. and housed in the animal facility with *ad libitum* access to food and water. The controlled environment was maintained at 23°C–25°C, 50%–55% humidity, and a 12 h light/12 h dark cycle. The rats were subjected to SCI and intraperitoneally injected with tomatidine (5, 10, or 20 mg/kg) for 7 days. Five groups, namely, control group, model group, model + 5 mg/kg tomatidine group, model + 10 mg/kg tomatidine group, and model + 20 mg/kg tomatidine, were constructed for the experiment. The rats were anesthetized with 50 mg/kg pentobarbital sodium, followed by cervical dislocation. Spinal cord injury tissues and serum were collected for further analysis. All animals were performed in accordance with an approved protocol by the Institutional Animal Care and Use Committee of Bestcell Model Biological Center (approval number: 2024-04–29A).

### 2.2 Cell culture and treatment

PC-12 cells were purchased from the American Type Culture Collection (Manassas, VA, United States), maintained in the DMEM medium (Gibco, Carlsbad, CA, United States) with 10% FBS (Gibco, United States) and 100 U/mL penicillin/streptomycin (Gibco, United States), and kept in the incubator at 37°C with a 5% CO_2_. PC-12 cells were stimulated with 100 ng/mL LPS (Sigma-Aldrich; Merck KGA) for 4 h to establish SCI model *in vitro*. After that, LPS-induced cells were treated with various concentrations of tomatidine (1 μM, 3 μM, or 10 μM), PMA, or CXCL10 inhibitor.

### 2.3 SCI model establishment

Rats were anesthetized by intraperitoneal injection of sodium pentobarbital (50 mg/kg), followed by laminectomy. In brief, the rats were fixed prone on the operating table, and the back was shaved and sterilized with iodine. A 2–3 cm long incision was made in the middle of the back, and the muscle and fascia were separated layer by layer to reveal the T10/T11 vertebrae. A 3-mm-long laminectomy was performed on the caudal end of the T10 vertebra and the cephalic end of the T11 vertebra under an anatomical body-viewing microscope. The observation of obvious congestion and swelling of the spinal cord and the contraction-like flutter and flaccid paralysis of both hind limbs suggested a successful injury. After the model was established, different concentrations of tomatidine were injected intraperitoneally into the rats for 7 consecutive days. Animal manipulation was approved by the Institutional Animal Protection and Use Committee of Nanjing University.

### 2.4 Behavioral assessment

The locomotor coordination function of rats at 0, 3, 7, 14, 21, and 28 days after SCI modeling was assessed by the blood-brain barrier (BBB) score, which ranges from 0 (complete paralysis) to 21 (normal locomotion). These standards are based on accurate observations of the lower extremities, including movement, pace, and coordinated motor action. The outcome value was determined by observing and scoring the average motor score of behaviors involving hind limbs.

### 2.5 Spinal cord water content evaluation

The water content was determined by the wet–dry weight method. For the determination of spinal cord water content, the spinal cord tissues collected at 7 days post-injury were weighed to obtain the wet weight (ww), and then the samples were dried at 100°C for 24 h and reweighed to obtain the dry weight (dw). The ratio of wet-to-dry weight was calculated using the following formula: $\left(\text{ww}-\text{dw}\right)/\text{ww}\times 100\%$ .

### 2.6 Hematoxylin and eosin staining

After the spinal cord tissues were fixed with 4% paraformaldehyde and dehydrated, 5 μm paraffin sections were made from the spinal cord plane of the injured site. The sections were dewaxed, hydrated, and incubated with hematoxylin (H9627-25G, Sigma) for 5 min. Then, the sections were stained with eosin (D12621, Xiya Reagent) at room temperature for 1–3 min. After that, the sections were dehydrated, cleared in anhydrous ethanol (100092680, Sinopharm Chemical Reagent Co., Ltd.) and xylene (10023418, Sinopharm Chemical Reagent Co., Ltd.), and sealed with neutral gum (10004160, Sinopharm Chemical Reagent Co., Ltd.). The morphology of spinal cord tissues was observed under a microscope (IX51, Olympus).

### 2.7 TUNEL analysis

Apoptotic cells in the spinal cord tissues were detected using the TUNEL kit (RCT-200R Ru Chuang Biology). In brief, the spinal cord tissues were fixed with 4% paraformaldehyde (80096618, Sinopharm Chemical Reagent Co., Ltd.) for 20 min, and 2–3 μm paraffin sections were taken after dehydration. After that, the sections were dewaxed in xylene for 15 min, washed with anhydrous ethanol for 5 min, soaked in 0.2% Triton X-100 (30188928, Sinopharm Chemical Reagent Co., Ltd.) for 15 min, and again soaked in DAPI solution (D8417-1MG, Sigma) for 20–30 min. Then, the slices were photographed and counted under an optical microscope (Eclipse Ci-L, Nikon).

### 2.8 Enzyme-linked immunosorbent assay

The rat serum samples were thawed and centrifuged at 4°C for 10 min. The insoluble substances were removed, and the supernatant was collected. Then, the inflammatory cytokines in the supernatant sample were quantified using the rat tumor necrosis factor (TNF)-α ELISA kit (ELK1396, ELK Biotechnology), rat IL-1β ELISA kit (ELK1272, ELK Biotechnology), rat IL-4 ELISA kit (ELK1154, ELK Biotechnology), rat IL-6 ELISA kit (ELK1158, ELK Biotechnology), rat IL-10 ELISA kit (ELK1144, ELK Biotechnology), and rat nerve growth factor ELISA kit (NGF, ELK2295, ELK Biotechnology), according to the manual. The OD value at 450 nm was determined on the Multiskan Spectrum (Cmax plus, Molecular) according to the manual.

### 2.9 Immunofluorescence staining

The spinal cord tissues were fixed in 4% paraformaldehyde (80096618, Sinopharm Chemical Reagent Co., Ltd.) for 20 min and supplemented with 0.2% Triton X-100 (30188928, Sinopharm Chemical Reagent Co., Ltd.) for 15 min. Subsequently, they were incubated with anti-CXCL10 antibody (10937-1-AP, 1:200, Proteintech) at 4°C overnight. After washing with PBS, the samples were hatched with goat anti-rabbit IgG HL Alexa Fluor 488 antibodies (ab150077, 1:500, Abcam) at 37°C for 30 min. DAPI solution (D8417-1MG, Sigma) was utilized to stain nuclei for 20–30 min, and images were obtained under a fluorescence microscope (Olympus, Japan).

### 2.10 Western blot assay

Total proteins from spinal cord tissues and PC-12 cells were lysed using the RIPA lysis buffer (AS1004, ASPEN) on ice for 30 min. Protein concentration was determined using the BCA Protein Quantification Kit (AS1086, ASPEN). Equal amounts of protein were mixed with SDS buffer, boiled for 5 min, fractionated by 12% SDS‒PAGE (AS1012, ASPEN), and transferred onto PVDF (IPVH00010, Millipore). After that, the membranes were blocked with 5% skimmed milk in TBST and cultivated overnight at 4°C with primary antibodies against p-p65, p65, cleaved-caspase 3, caspase 3, CXCL10, and GAPDH (1:1,000, ASPEN). After washing three times with TBST, the membranes were incubated with secondary antibodies for 30 min and detected using the Novex™ ECL Chemiluminescent Reagent Kit (AS1059, ASPEN). The relative protein expression was detected using AlphaEaseFC software.

### 2.11 Quantitative real time-polymerase chain reaction

Total RNA was extracted from spinal cord tissues and PC-12 cells using the TRIpure Total RNA Extraction Reagent (EP013, ELK Biotechnology), following the instructions, and was reverse-transcribed into cDNA using an EntiLink™ 1st Strand cDNA Synthesis Super Mix (EQ031, ELK Biotechnology). Then, the EnTurbo™ SYBR Green PCR SuperMix (EQ001, ELK Biotechnology) with the QuantStudio 6 Flex System PCR (Life Technologies) was applied for quantitative real time-polymerase chain reaction (qRT-PCR). Reactions were carried out under the following cycling conditions: an initial denaturation at 95°C for 30 s, followed by 40 cycles for 10 s at 95°C, 30 s at 58°C, 30 s at 72°C, and a final extension at 55°C for 11 min. *GAPDH* was regarded as the reference gene. After that, the relative mRNA expression levels were calculated using the 2^−ΔΔCT^ method. The following primer sequences were used: CXCL10 (forward: 5′-CTG​AGT​GGG​ACT​CAA​GGG​ATC -3′; reverse: 5′-TTC​AGA​CAC​CTC​TTC​TCA​TTG​TTC-3′) and GAPDH (forward: 5′-GCC​AAG​GTC​ATC​CAT​GAC​AAC-3′; reverse: 5′-GTG​GAT​GCA​GGG​ATG​ATG​TTC-3′).

### 2.12 Cell counting Kit-8 assay

Cell Counting Kit-8 (C0037, Beijing, China) was applied for cell viability evaluation. PC-12 cells (1 × 10^4^/well) were seeded into 96-well plates for 24 h. Next, the cells were stimulated with 10 μL of the CCK-8 reagent and incubated at 37°C for 2 h. A microplate reader (Molecular Devices, United States) was used to determine the absorbance at a 450 nm wavelength.

### 2.13 Flow cytometry analysis

PC-12 cells (5 × 10^4^ cells/mL) were digested with trypsin, washed using PBS, and centrifuged at 1,200 rpm for 5 min. After that, the apoptotic rate of neuronal cells was determined using the FITC Annexin V Apoptosis Detection Kit (C1062L, Beyotime), following the manufacturer’s instructions. Then, the cells were estimated via flow cytometry (BD Biosciences, San Jose, CA).

### 2.14 Statistical analysis

Data were given as the means ± standard deviation (SD). Comparisons between two groups were assessed using the Student’s *t*-test, and differences among multiple groups were analyzed using a one-way ANOVA, followed by Tukey’s *post hoc* test. All experiments were conducted in triplicate. All statistical analyses were performed using GraphPad Prism 8.0 software. A *p*-value < 0.05 was considered statistically significant.

## 3 Results

### 3.1 Tomatidine promoted the recovery of SCI rats through inhibiting neuronal apoptosis and inflammatory response

**FIGURE 1:**
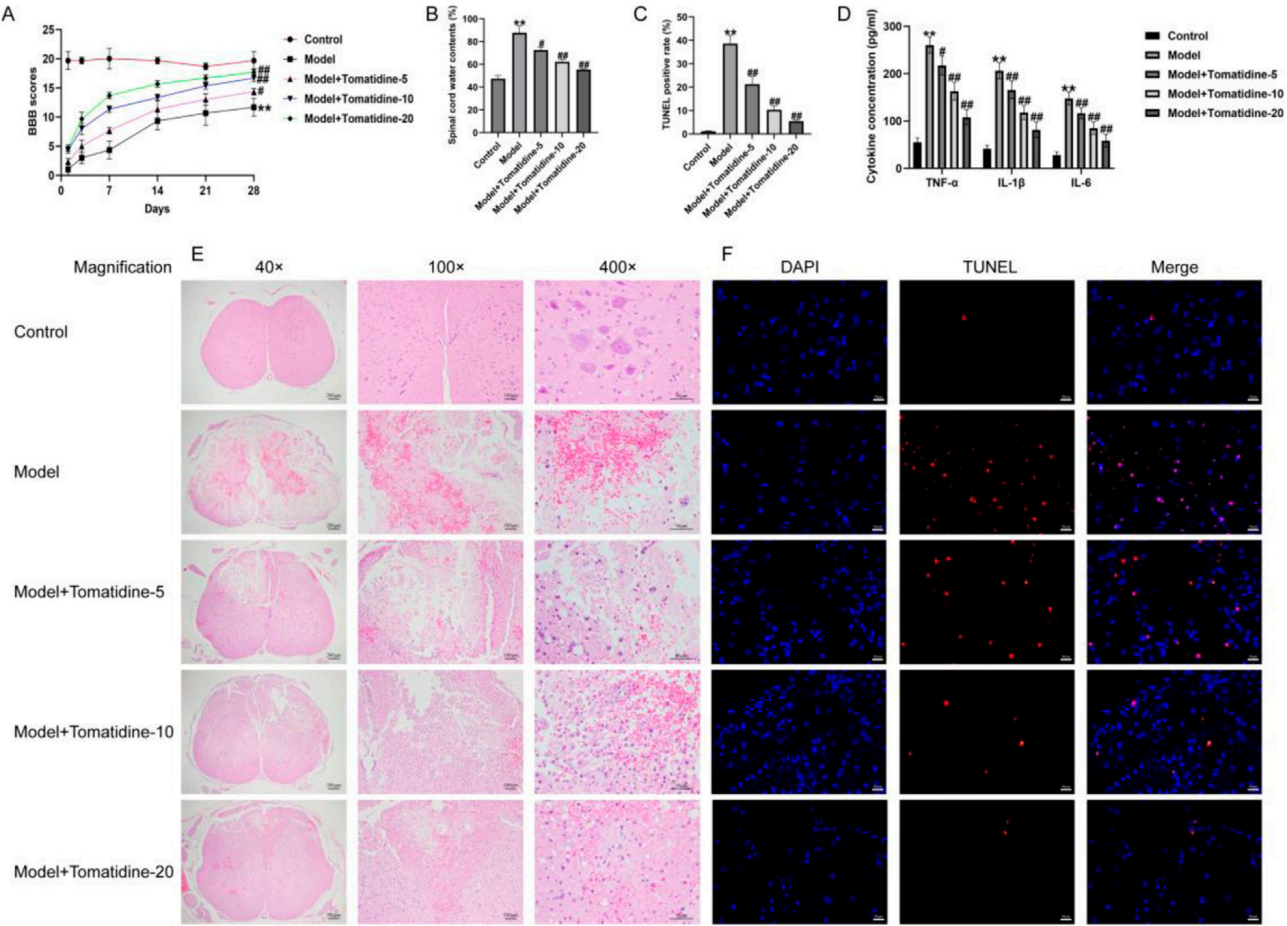
Tomatidine alleviated SCI by suppressing neuron apoptosis and inflammatory responses. The rats were subjected to SCI and intraperitoneally injected with tomatidine (5, 10, or 20 mg/kg) for 7 days. Rats were divided into five groups: control, model, model + 5 mg/kg tomatidine, model + 10 mg/kg tomatidine, and model + 20 mg/kg tomatidine groups. **(A)** BBB scores at 0, 7, 14, 21, and 28 days after SCI were calculated. **(B)** Spinal cord water content was evaluated by the wet-to-dry weight method. **(C)** Quantification of the TUNEL-positive rate. **(D)** Levels of TNF-α, IL-1β, and IL-6 in serum of rats were determined using ELISA assays. **(E)** Pathological injury of spinal cord tissues was evaluated using H&E staining. Scale bar: 50, 100, and 200 μm. **(F)** TUNEL staining was conducted to evaluate the apoptotic cells in spinal cord tissues. Scale bar: 20 μm. **(A–D)** n = 6/group. *p < 0.05 and **p < 0.01 *vs*. control group; ^#^ *p* < 0.05 and ^##^ *p* < 0.01 *vs*. model group.

First, we established a rat model of SCI and evaluated the roles of tomatidine in SCI, and the behavioral analyses were determined. As presented in Figure 1A, the model group showed lower BBB scores at 7, 14, 21, and 28 h compared with the control group. Moreover, the water contents in spinal tissues were elevated in the model group, by contrast with the control group (Figure 1B). H&E staining suggested that the spinal cord tissue structure of rats in the control group was integrated, with no destructive cells and inflammatory infiltration. However, we observed serious structural damage to the spinal cord tissue in the SCI group, with cell cracks and other serious pathological damage (Figure 1E). In addition, the TUNEL staining assay revealed that the proportion of TUNEL-positive cells in the SCI group was higher than that in the control group (Figures 1C, F). These observations indicated that SCI rat models were successfully constructed. Our results further demonstrated that tomatidine treatment partially reversed the SCI injury, as confirmed by obviously increased BBB scores, reduced water content, relieved the structural damage, and inhibition of TUNEL positive cell percentage in SCI rats in a dose-dependent manner. In addition, the secretion of pro-inflammatory factors, including TNF-α, IL-1β, and IL-6, was further determined using the ELISA assay. Our data suggested that the pro-inflammatory factor levels were significantly enhanced in the spinal cord tissues of SCI rats in a dose-dependent manner, as opposed to the control group, while tomatidine treatment exhibited reversed effects (Figure 1D). Taken together, our results indicated that tomatidine promoted the recovery of nerve function by reducing SCI-induced neuronal apoptosis and inflammatory response in SCI.

### 3.2 Tomatidine promoted the recovery of spinal cord tissues by regulating CXCL10 expression in SCI rats

**FIGURE 2:**
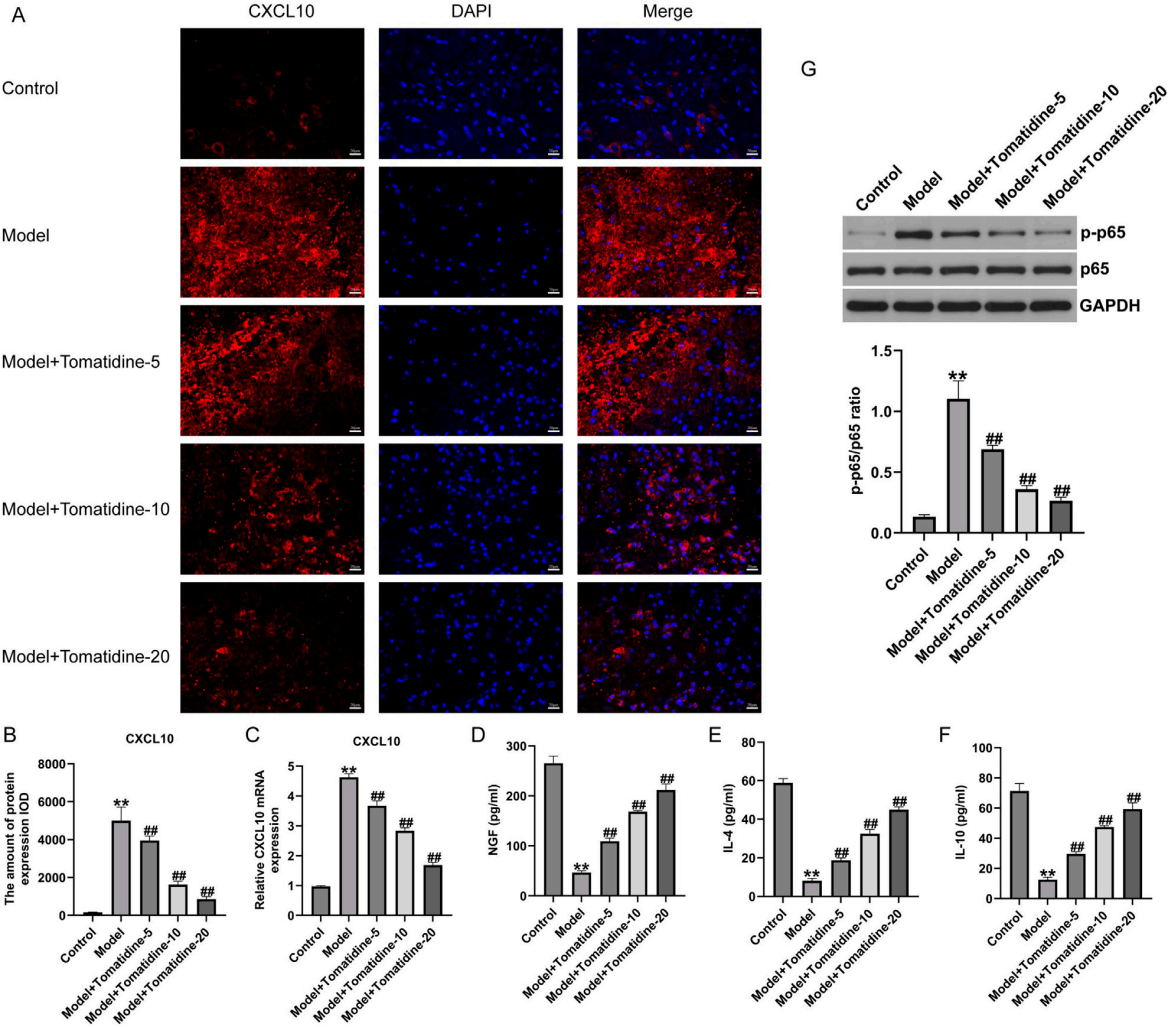
Tomatidine reversed the CXCL10 expression and inhibited the NF-κB pathway in the spinal cord tissues of SCI rats. **(A)** Immunofluorescence staining was used to evaluate the CXCL10 expression. Scale bar: 20 μm. **(B)** Quantification of CXCL10 expression. **(C)** CXCL10 mRNA expression was measured by qRT-PCR analysis. The levels of NGF **(D)**, IL-4 **(E)**, and IL-10 **(F)** in serum of rats were determined using ELISA assays. **(G)** Protein expression level and quantification of p-p65 and p65 in spinal cord tissues of rats. **(B–F)**: n = 6/group; **(G)** n = 3/group *p < 0.05 and **p < 0.01 *vs*. control group; ^#^ *p* < 0.05 and ^##^ *p* < 0.01 *vs*. model group.

To investigate the role of CXCL10 in SCI, we determined the expression of CXCL10 in the spinal cord tissues using immunofluorescent staining and qRT-PCR analysis. As illustrated in Figures 2A, B, the expression of CXCL10 in the model group was remarkably higher than that in the control group. The introduction of tomatidine led to the remarkable downregulation of CXCL10 in spinal cord tissues in a dose-dependent manner. qRT-PCR results, in line with immunofluorescent staining findings, revealed that CXCL10 was overexpressed in the spinal cord tissues of SCI rats; however, this overexpression was inhibited in a dose-dependent manner after tomatidine treatment (Figure 2C). The serum NGF level was significantly decreased in SCI rats compared with control rats. Tomatidine treatments significantly increased serum NGF levels in a dose-dependent manner (Figure 2D). The levels of IL-4 and IL-10 were decreased in SCI rats compared to control rats, and these anti-inflammatory markers were significantly increased in a dose-dependent manner after tomatidine treatments in SCI rats (Figures 2E, F). Our findings suggested that tomatidine relieved neuronal injury in SCI through regulating CXCL10 expression and anti-inflammatory response.

### 3.3 Tomatidine inhibited the activation of the NF-κB pathway in SCI rats

Increasing the number of reports has revealed that the NF-κB pathway is a vital signaling pathway related to CXCL10 synthesis in nervous injury models (Nie et al., 2022). Next, we assessed whether tomatidine affects the NF-κB pathway activation in SCI, and a Western blot assay was conducted to determine the NF-κB pathway-associated gene expression. We observed that p-p65 was upregulated in SCI rats in comparison with the control group. However, after stimulation with tomatidine, the expression level of p-p65 was downregulated in a dose-dependent manner relative to the model group (Figure 2G). Our data demonstrated that tomatidine blocked the activation of the NF-κB pathway in SCI.

### 3.4 PMA enhanced the CXCL10 expression and activated the NF-κB pathway in tomatidine-treated SCI rats

**FIGURE 3:**
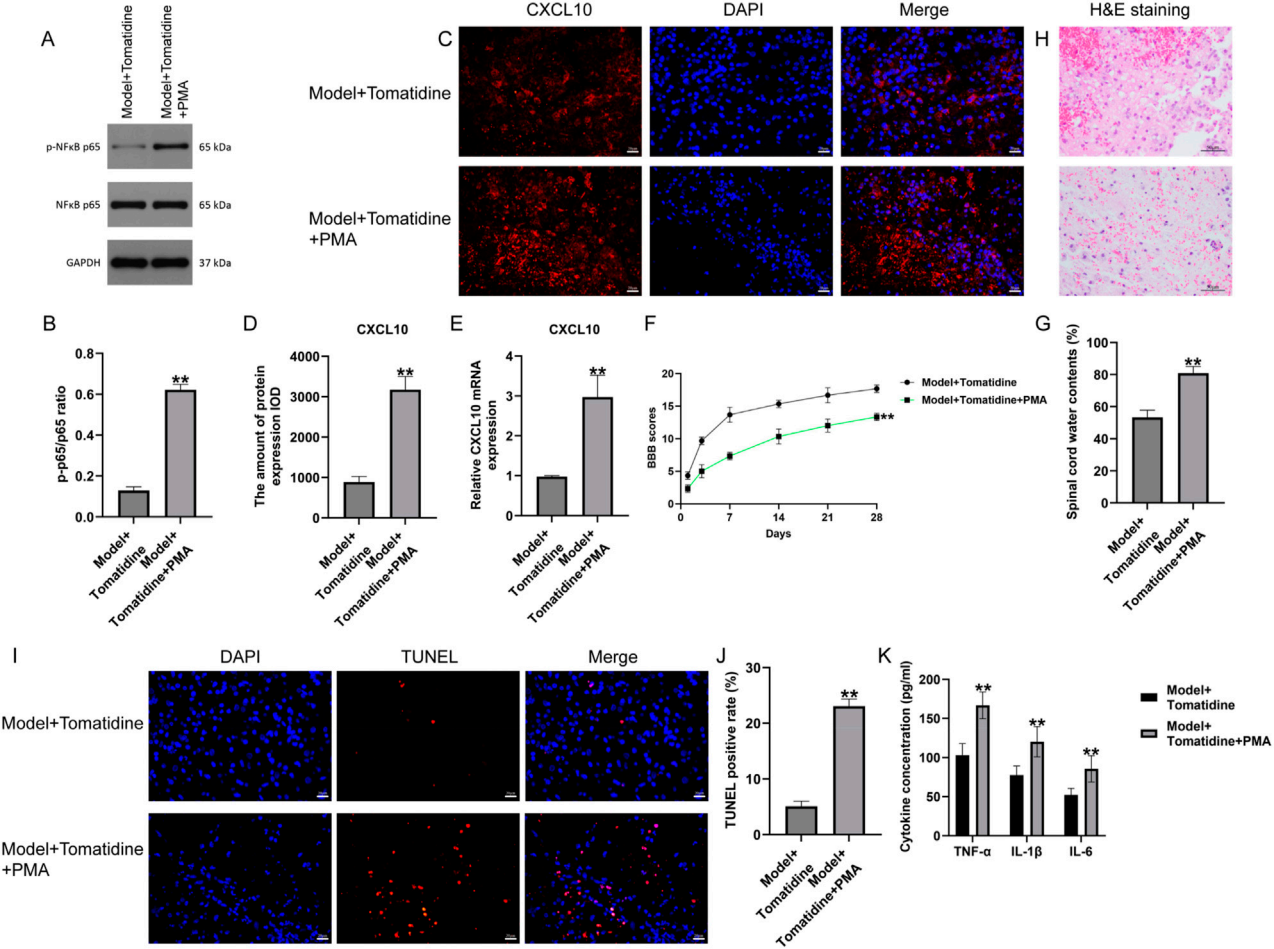
Effects of tomatidine on CXCL10 expression, NF-κB pathway, neurological function recovery, and neuron apoptosis in rats. **(A)** Protein expression level of p-NF-κB p65. **(B)** Quantification of the p-P65/P65 ratio. **(C)** CXCL10 expression was determined using immunofluorescence staining. Scale bar: 20 μm. **(D)** Quantification of CXCL10 expression. **(E)** qRT-PCR analysis of the CXCL10 mRNA level. **(F–K)** Rats were subjected to SCI and intraperitoneally injected with tomatidine or tomatidine + NF-κB pathway agonist. Rats were divided into two groups: model + tomatidine group and model + tomatidine + PMA group. **(F)** The BBB scores at 0, 7, 14, 21, and 28 days after SCI were calculated. **(G)** Spinal cord water content was evaluated by the wet-to-dry weight method. **(H)** Histopathological changes in the spinal cord tissues of rats were determined using H&E staining. Scale bar: 50 μm. **(I)** Apoptotic cells in spinal cord tissues were determined using TUNEL staining. Scale bar: 20 μm. **(J)** Quantification of the TUNEL-positive rate. **(K)** Levels of TNF-α, IL-1β, and IL-6 in serum of rats were determined using ELISA assays. **(B, D, J).** n = 3/group; **(E–G, K)** n = 6/group. *p < 0.05 and **p < 0.01 *vs*. model + tomatidine group.

To further investigate the roles of tomatidine in the regulation of the NF-κB pathway, the pathway agonist (PMA) was applied to activate this pathway in tomatidine-treated SCI rats. As exhibited in Figures 3A, B, the Western blot assay indicated that p-p65 expression was dramatically elevated in the spinal cord tissues of SCI rats post tomatidine + PMA treatment. Furthermore, immunofluorescent staining and qRT-PCR analysis were conducted to investigate the CXCL10 expression. We found that CXCL10 expression was remarkably upregulated in the spinal cord tissues of tomatidine + PMA treated SCI rats, as opposed to that in the tomatidine alone treated group (Figures 3C–E). Collectively, PMA reversed the protective effects of tomatidine on the CXCL10/NF-κB pathway in SCI.

### 3.5 PMA suppressed the neurological function recovery and promoted neuron apoptosis and inflammation in tomatidine-treated SCI rats

To further explore the regulatory mechanism of PMA in tomatidine-treated SCI rats, the effects of PMA on neurological function were evaluated. Our data suggested that PMA treatment reduced BBB scores at 7, 14, 21, and 28 days, as well as enhanced the water contents in spinal tissues, compared with the tomatidine-treated SCI rats (Figures 3F, G). Moreover, H&E staining revealed that the injury area and inflammatory cell infiltration were increased in tomatidine + PMA-treated SCI rats compared to the tomatidine alone group (Figure 3H). Further analysis using TUNEL staining and ELISA assays demonstrated that PMA increased the proportion of TUNEL-positive cells (Figures 3I, J) and promoted the secretion of inflammatory factors (Figure 3K) compared with the tomatidine group. In summary, PMA reversed the protective effects of tomatidine on neurological function recovery, neuron apoptosis, and inflammation.

### 3.6 Tomatidine relieved neuronal apoptosis and inflammation in LPS-induced PC-12 cells by regulating CXCL10 expression

**FIGURE 4:**
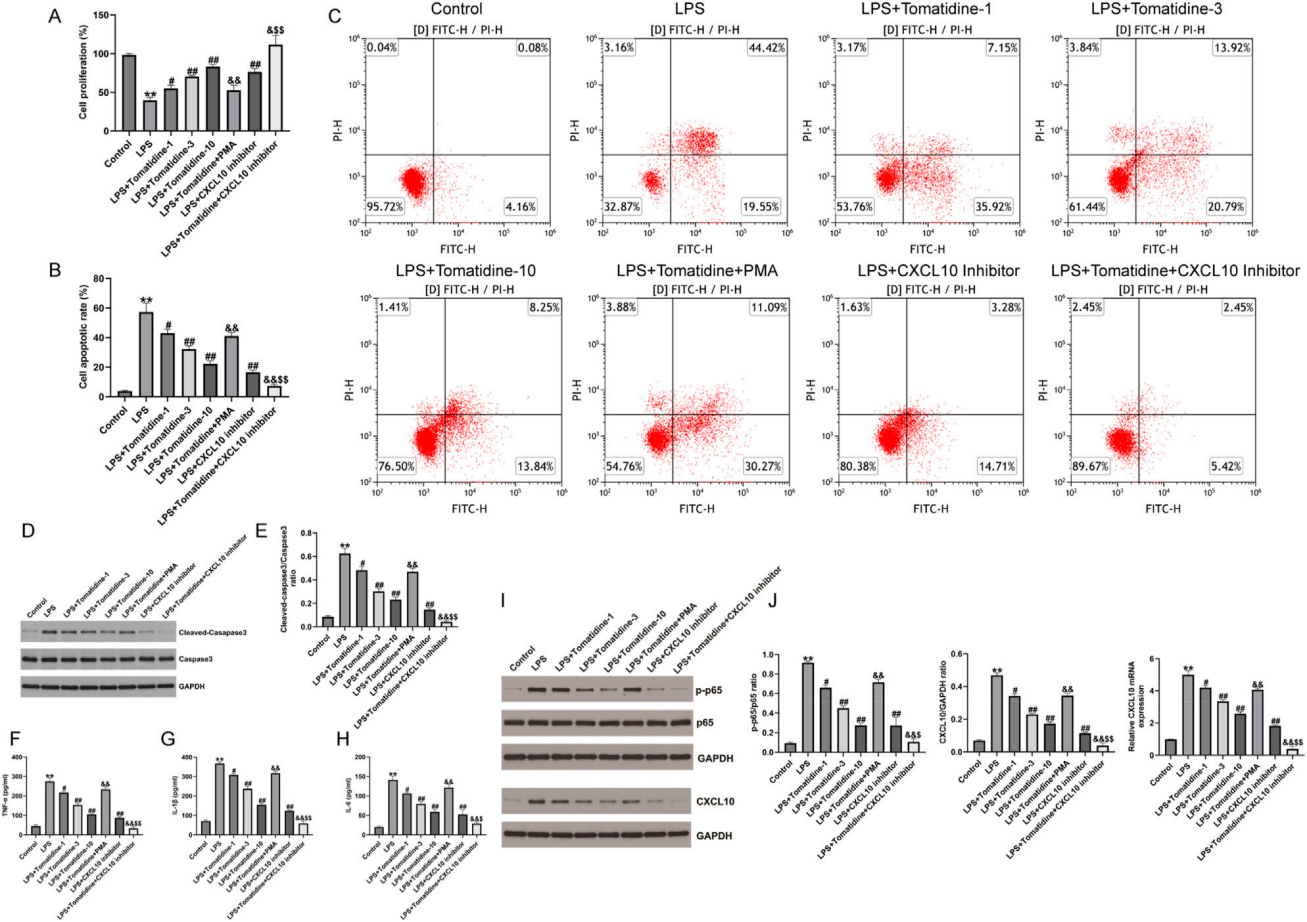
Effects of tomatidine, PMA, and CXCL10 inhibitor on LPS-induced neuronal proliferation, apoptosis, inflammation, CXCL10 expression, and NF-κB pathway in PC-12 cells. PC-12 cells were induced by 100 ng/mL LPS for 4 h and treated with various concentrations of tomatidine (1 μM, 3 μM, or 10 μM), PMA, or a CXCL10 inhibitor. Cells were divided into eight groups: control, LPS, LPS + 1 μM tomatidine, LPS + 3 μM tomatidine, LPS + 10 μM tomatidine, LPS + 10 μM tomatidine + PMA, LPS + CXCL10 inhibitor, and LPS + 10 μM tomatidine + CXCL10 inhibitor group. **(A)** Cell viability was determined using the CCK-8 assay. **(B)** Quantification of the cell apoptotic rate. **(C)** Cell apoptosis was evaluated by flow cytometry. **(D)** Western blot analysis of cleaved caspase 3 and caspase 3 in PC-12 cells. **(E)** Quantification of cleaved caspase 3/caspase 3 ratio. The levels of TNF-α **(F)**, IL-1β **(G)**, and IL-6 **(H)** in serum of rats were determined using ELISA assays. **(I)** Protein expression level of p-p65 and CXCL10. **(J)** Quantification of the p-p65/p65 ratio, CXCL10 expression, and CXCL10 mRNA level (n = 3/group). *p < 0.05 and **p < 0.01 *vs*. control group; ^#^ *p* < 0.05 and ^##^ *p* < 0.01 *vs*. LPS group; ^&^ *p* < 0.05 and ^&&^ *p* < 0.01 *vs*. LPS + 10 μM tomatidine group; ^$^ *p* < 0.05 and ^$$^ *p* < 0.01 *vs*. LPS + CXCL10 inhibitor.

In order to illustrate the mechanism associated with the protection of tomatidine against SCI in the cell model, we constructed SCI cell models using 100 ng/mL LPS-treated PC-12 cells for 4 h. After that, PC-12 cells were treated with various concentrations of tomatidine (1 μM, 3 μM, and 10 μM). As displayed in Figures 4A–C, LPS inhibited cell proliferation and promoted cell apoptosis in LPS-treated cells, which were partly reversed by tomatidine in a dose-dependent manner. Moreover, tomatidine remarkably decreased LPS-stimulated cleaved-caspase-3 expression (Figures 4D, E) and reduced the secretion of inflammatory factors (Figures 4F–H) in PC-12 cells in a dose-dependent manner. Next, the effects of NF-κB agonist PMA and CXCL10 inhibitor on neuronal proliferation and apoptosis were verified. Our data further revealed that the cell proliferation was decreased, apoptotic cells were enhanced, cleaved-Caspase-3 expression was upregulated, and inflammatory response was elevated in the groups exposed to PMA irradiation. In contrast, the treatment of the CXCL10 inhibitor elevated cell proliferation and decreased the apoptotic cells, cleaved-Caspase-3 expression, and inflammatory response in LPS-induced PC-12 cells, and the effects of the CXCL10 inhibitor were further enhanced by tomatidine treatment. Taken together, tomatidine protected neurons against LPS-induced injury in PC-12 cells by regulating CXCL10.

### 3.7 Tomatidine relieved neuronal damage in LPS-induced PC-12 cells by regulating the NF-κB/CXCL10 pathway

We also assessed whether tomatidine relieved neuronal damage in LPS-induced PC-12 cells by regulating the NF-κB/CXCL10 pathway in SCI, and Western blot was conducted to determine the NF-κB/CXCL10 pathway-associated gene expression. We observed that p-p65 and CXCL10 were upregulated in LPS-induced PC-12 cells in comparison to the control group. However, after stimulation with tomatidine, the expression levels of p-p65 and CXCL10 were downregulated in a dose-dependent manner relative to the model group (Figures 4I, J). In addition, tomatidine inhibited the relative mRNA levels of CXCL10 in LPS-induced PC-12 cells in a dose-dependent manner (Figures 4I, J). However, we observed the opposite results in PMA-treated cells. In contrast, the treatment of the CXCL10 inhibitor reduced the activation of the NF-κB pathway and decreased the CXCL10 expression in LPS-induced PC-12 cells, and the inhibitory effects of the CXCL10 inhibitor were further enhanced by tomatidine treatment. Taken together, tomatidine protected neurons against LPS-induced injury in PC-12 cells by regulating the NF-κB/CXCL10 pathway.

## 4 Discussion

SCI represents a prevalent cause of disability among young adults. Achieving independent ambulation in these patients is regarded as a significant challenge within the field of regenerative medicine as the repair of spinal cord injury necessitates intricate processes, including axonal regeneration, remyelination, and the establishment of new synaptic connections (Ben-Hur, 2010; Venkatesh et al., 2019). Research studies have revealed that multiple molecular events are involved in the development of SCI, including neuronal apoptosis, oxidative stress, and inflammation (Ma et al., 2023). At present, the clinical treatment of SCI includes surgical intervention and high-dose methylprednisolone shock therapy (Duan et al., 2021). However, these treatments can improve the overall survival rate of patients but cannot repair damaged neurological function. In recent years, research has been conducted on the pathological process and potential mechanisms of SCI, but strategies for SCI are still limited. In this study, we constructed SCI models *in vivo* and *in vitro* and revealed potential molecular mechanisms of tomatidine involved in the progression of SCI. Our data revealed that tomatidine alleviated neuronal damage by suppressing SCI-induced apoptosis and inflammatory responses through blocking NF-κB/CXCL10 pathway activation.

Tomatidine, a natural steroidal alkaloid, is related to inflammatory responses in many diseases. Chu et al. (2020) revealed that tomatidine inhibited IL-1β-induced inflammation in primary chondrocytes through the NF-κB pathway in osteoarthritis. Xu et al. (2024) suggested that tomatidine activated autophagy to improve lung injury and inflammation in sepsis by suppressing NF-κB and MAPK pathways. However, whether tomatidine improves inflammation in the progression of SCI remains unknown. In this research, the intervention of tomatidine resulted in an obvious reduction in the secretion of pro-inflammatory cytokines (IL-1β, IL-6, and TNF-α) in the spinal cord tissues of SCI rats, implying that tomatidine may alleviate SCI by inhibiting inflammation. Apoptosis is a vital process that affects the progression of SCI-induced neuronal tissue damage. Tan et al. (2021) found that the p75 neurotrophin receptor enhanced SCI-induced neuronal apoptosis by downregulating NTRK3. Moreover, He et al. (2021) revealed that the selective NLRP3 inhibitor MCC950 reduced neuronal apoptosis and promoted the recovery of motor function in SCI. In the present study, we found that tomatidine mitigated SCI with increased BBB score, decreased spinal cord water content, reduced the percentage of TUNEL-positive cells, and improved histopathological lesions in a dose-dependent manner. In line with the *in vivo* results, we also found that tomatidine decreased the cleaved-caspase 3 expression in the LPS-induced PC-12 cell injury model, indicating that tomatidine has a protective effect on SCI by inhibiting neuronal apoptosis and the inflammatory response. However, the protective roles of tomatidine against SCI require further investigation.

Inflammatory factors include a variety of cytokines and chemokines. A growing number of research studies have illustrated that CXCL10 was involved in the regulation of neuronal damage after SCI (Mordillo-Mateos et al., 2019). However, the role of tomatidine on CXCL10 in SCI has not been reported. The results of this research suggested that the expression of CXCL10 was memorably elevated after SCI in rats and LPS-induced PC-12 cells, while tomatidine treatment decreased the CXCL10 expression in a dose-dependent manner. As a key signal transducer, NF-κB plays an important role in the inflammatory response induced by inflammatory cytokines and chemokines (Yu H. et al., 2020). NF-κB, a main regulator of inflammation, is involved in the occurrence and development of SCI. For example, Liu et al. (2021) revealed that SARM1 promotes neuroinflammation and suppresses neural regeneration after SCI through the NF-κB pathway. Moreover, Zhao et al. (2021) suggested that resveratrol, a stilbene molecule belonging to the polyphenol family, achieved neuroprotective effects by suppressing inflammation regulated by the NF-κB pathway in SCI. Our investigation is consistent with these results, suggesting that tomatidine blocked the NF-κB pathway activation in SCI, as evidenced by reduced P-p65 expression. Thus, the inhibition of NF-ĸB pathway activation may be a potential treatment for SCI.

Previous studies have suggested that PMA binds to specific cell surface receptors of macrophages, which eventually leads to the phosphorylation and activation of the NF-κB pathway and the secretion of inflammatory factors (Lei et al., 2023). In addition, PMA activates protein kinase C-epsilon (PKCε) in PC-12 cells by binding to the C1 domain. Zhang et al. (2023) revealed that the NF‐κB pathway promoter PMA reversed the beneficial effects of PBM on SCI by regulating CXCL10 expression. Our results demonstrated that PMA reversed the protective effect of tomatidine on spinal cord injury, which can attributed, at least in part, to the following mechanisms: tomatidine inhibited neuronal damage in the SCI, resulting in a reduction in the NF-ĸB phosphorylation level, which further decreased the chemokine CXCL10 expression, suppressed the neuronal apoptosis and inflammatory response, and inhibited the occurrence of SCI. Furthermore, the CXCL10 inhibitor was used to further explore the regulatory roles of CXCL10 in SCI. We also found that the CXCL10 inhibitor inhibited the activation of the NF-κB pathway and decreased the CXCL10 expression in LPS-induced PC-12 cells, and the inhibitory effects of the CXCL10 inhibitor were further strengthened by tomatidine treatment, indicating that tomatidine may be a promising agent for blocking the NF-ĸB pathway in SCI.

The present study has several limitations that need to be addressed in future studies. Currently, various other phytochemicals, such as (-)-epigallocatechin-3-gallate (Álvarez-Pérez et al., 2016), curcumin (Zhang et al., 2017), resveratrol (Hu et al., 2017), and quercetin (Jiang et al., 2016), have been documented to exhibit protective effects against SCI. In the referenced animal studies, these phytochemicals were administered at doses ranging from 30 to 200 mg/kg of animal body weight. In contrast, the effective dose of tomatidine utilized in this study was between 5 and 20 mg/kg of animal body weight. These findings suggest that tomatidine may possess greater efficacy in safeguarding against SCI; however, further investigation is warranted to substantiate this potential. Pro-survival signaling networks are re-organized during the acute phase response to SCI. Several of these pro-survival signaling pathways may be adaptive and contribute to cellular survival following SCI. Notable examples include the activation of the ERK1/2-NF-κB signaling by IL-17B (Bastid et al., 2020), the activation of protein kinase B (Akt) via mechanistic target of rapamycin (mTOR) (Pegoraro et al., 2015; Neinast et al., 2019), or the protein tyrosine phosphatase non-receptor type 5 (PTPN5)-mediated dephosphorylation of ERK1/2 (Chu et al., 2004); however, the specific pro-survival signaling mechanisms underlying the beneficial effects of tomatidine on SCI remain to be elucidated.

**FIGURE 5:**
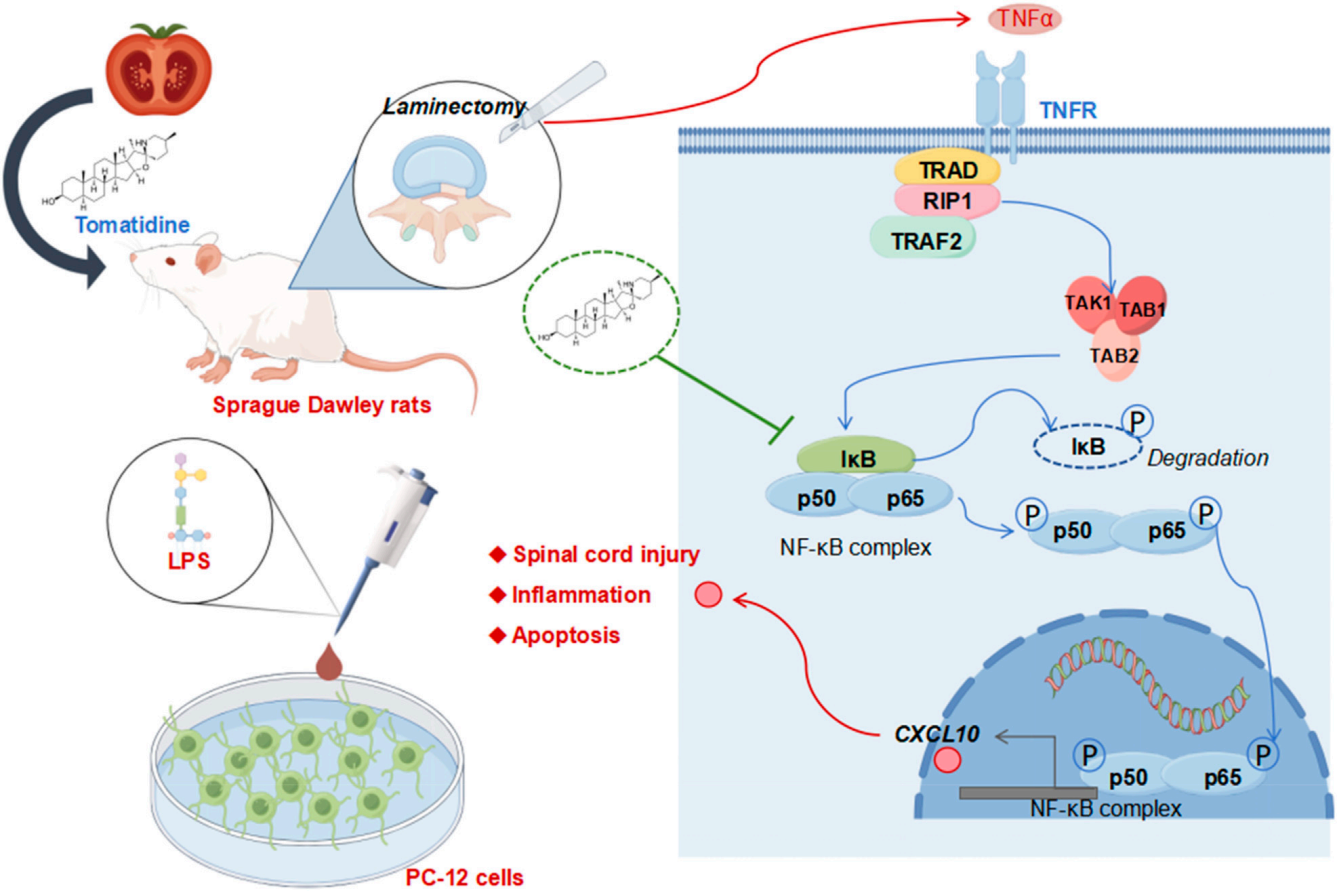
Overview of potential mechanisms of action of tomatidine in protecting against spinal cord injury.

Taken together, the *in vivo and in vitro* results of this study indicated that tomatidine improved neuronal damage in spinal cord injury by inhibiting the inflammatory responses and apoptosis through the NF-κB/CXCL10 pathway (Figure 5). Our findings may provide a novel perspective and a potential candidate for SCI treatment. However, other mechanisms underlying the regulation of SCI by tomatidine require further investigation. In addition, additional target genes capable of inhibiting the activation of the NF-ĸB pathway in SCI need to be further explored.

## Data Availability

The raw data supporting the conclusions of this article will be made available by the authors, without undue reservation.
